# Altered biphasic serotonin discharge hypothesis in mild traumatic brain injury

**DOI:** 10.2217/cnc-2021-0001

**Published:** 2021-07-02

**Authors:** Jun Shinoda, Hirohito Yano, Noriyuki Nakayama

**Affiliations:** 1 Chubu Medical Center for Prolonged Traumatic Brain Dysfunction, Kizawa Memorial Hospital, Minokamo, Japan; 2 Department of Clinical Brain Sciences, Gifu University Graduate School of Medicine, Minokamo, Japan; 3 Department of Neurosurgery, Gifu University Graduate School of Medicine, Gifu, Japan

**Keywords:** ^18^F-FDG-PET, concussion, dorsal raphe nucleus, head finite element model, mild TBI, post-concussion syndrome, serotonin

Mild traumatic brain injury (TBI) is the most common type of TBI, and about 30% of patients with mild TBI suffer from symptomatic sequelae for several months to years as post-concussion syndrome. Among the symptoms experienced, mental and cognitive sequelae are particularly intractable.

In recent years, advances in sophisticated MRI techniques have made it possible to visualize small traumatic lesions in living patients with diffuse brain injury (DBI). Diffusion tensor imaging (DTI) has been recognized as the most sensitive modality for detecting white matter injury in DBI. However, in a systematic review of DTI in patients with post-concussion syndrome, 30 and 50% of the reviewed studies showed no significant changes in fractional anisotropy and mean diffusivity, respectively [1]. In another review of DTI, there was a lack of complete agreement on the location and number of affected white matter tracts across different studies of patients with mild TBI [2]. Thus, injury of the white matter in mild TBI remains obscure, even with DTI. Why are the lesions causing symptoms in mild TBI not consistently visible on neuroimaging? We may need to postulate another pathological mechanism apart from the structural lesions shown on MRI as the cause of symptoms in mild TBI.

## Brain strain in mild TBI based on injury analysis of a finite element model

The acute phase of a concussion is characterized by a brief loss of consciousness. The force of impact may functionally disrupt normal cellular activity in the reticular activating system in the brainstem. Additionally, vulnerability of the upper brainstem may contribute to disturbances in the pupillary dynamics, accommodation and vergence movements often seen in patients with mild TBI. One likely pathology contributing to these symptoms is damage to the oculomotor and Edinger–Westphal nuclei, both of which are located in the midline structure of the midbrain.

**Figure 1.**
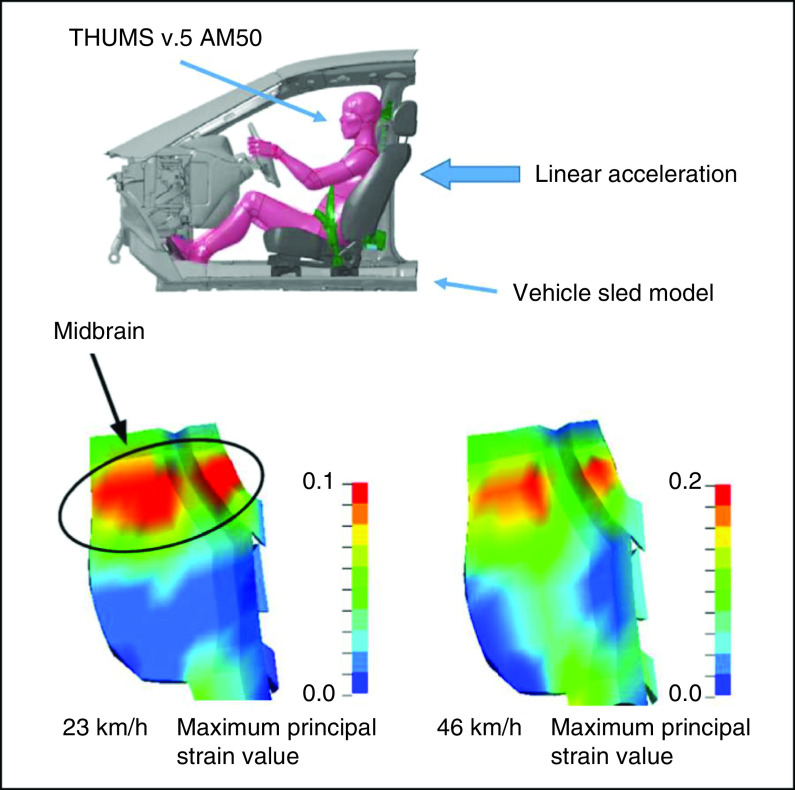
Computational injury analysis of collision studies using the finite element model. A Total Human Model for Safety (v.5) 50th percentile adult male occupant model was placed in the automotive seat of a vehicle sled model with a three-point seat belt. Comparisons of the maximum principal strain distribution between 23 km/h (86 ms after the onset of linear acceleration) and 46 km/h (82 ms after the onset of linear acceleration) in a rear-end collision at the sagittal section of the brainstem are shown. AM50: 50th percentile adult male; THUMS: Total Human Model for Safety. Reproduced with permission from [3] © Taylor & Francis (2020).

**Figure 2.**
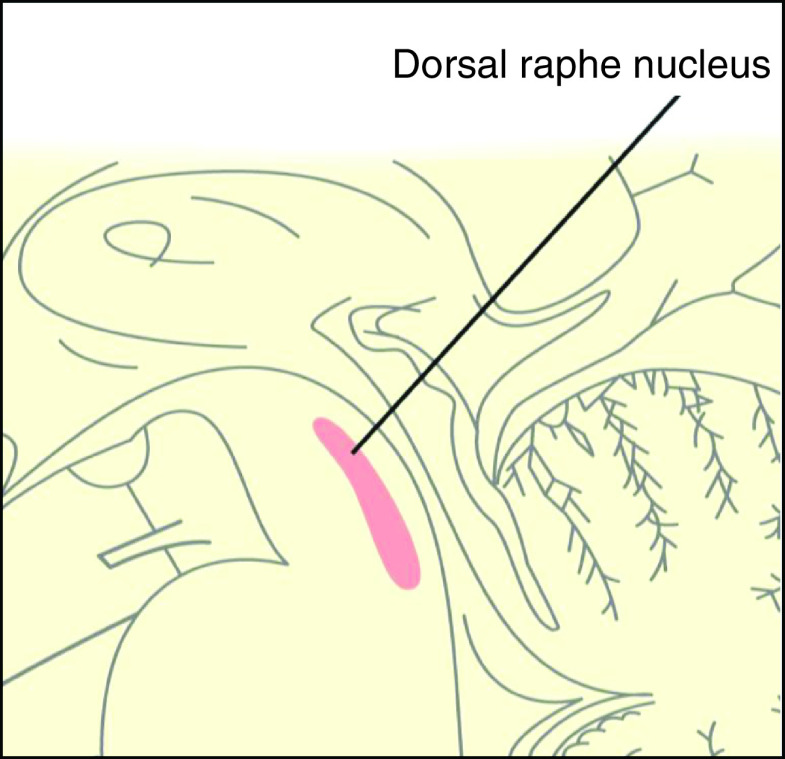
Dorsal raphe nucleus.

In our recent computational collision injury study using a human head finite element model, which simulates whiplash-associated injury, comparatively higher maximum principal shearing strain values were shown in the corpus callosum and dorsal midbrain (Figure 1) [3]. The results support this pathophysiology as the cause of loss of consciousness and visual dysfunction in mild TBI. The dorsal raphe nucleus (DRN) is also a midline structure of the dorsal midbrain and is located very near the oculomotor and Edinger–Westphal nuclei (Figure 2). According to the collision injury analysis, the DRN is likely highly affected in mild TBI as well. Damage to the DRN may just affect the appearance of symptoms in mild TBI.

## Glucose metabolism abnormalities in mild TBI

**Figure 3.**
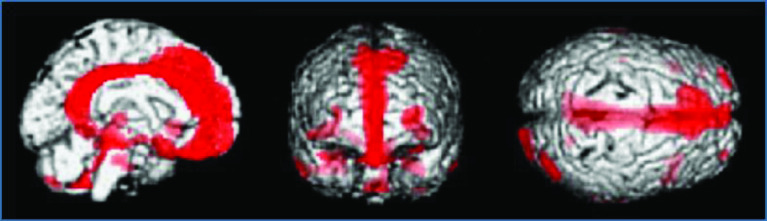
^18^F-fluorodeoxyglucose positron emission tomography in a patient group with chronic disorders of consciousness (minimally conscious and vegetative states) due to severe traumatic diffuse brain injury. The brain regions in which ^18^F-fluorodeoxyglucose uptake was significantly decreased (red) in the patient group compared with normal control subjects (p < 0.001) are shown. Reproduced with permission from [4] © BMJ Publishing Group (2006).

**Figure 4.**
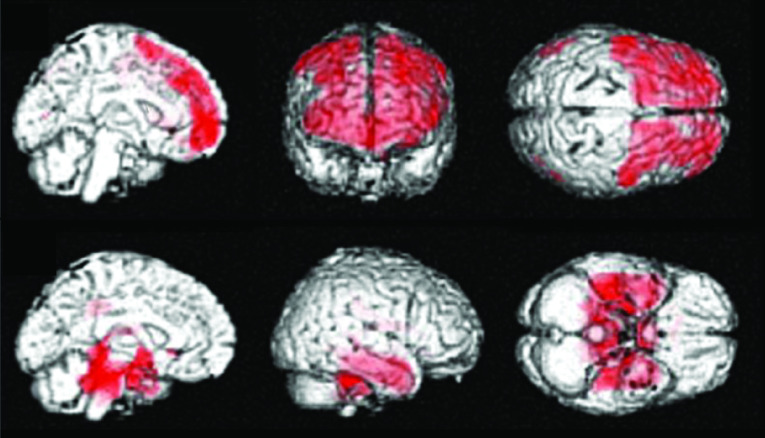
^18^F-fluorodeoxyglucose positron emission tomography in a patient group with chronic mental and cognitive symptoms due to mild traumatic brain injury without visible brain lesions. The brain regions in which ^18^F-fluorodeoxyglucose uptake was significantly decreased (red) in the patient group compared with normal control subjects (p < 0.05) (upper) and brain regions in which ^18^F-fluorodeoxyglucose uptake was significantly increased (red) in the patient group compared with normal control subjects (p < 0.05) (lower) are shown. Reproduced with permission from [5] © Mary Ann Liebert, Inc. (2019).

In patients with severe traumatic DBI corresponding to diffuse axonal injury (DAI), hypometabolic regions detected by ^18^F-fluorodeoxyglucose PET (^18^F-FDG-PET) have been consistently reported in the cingulum, medial prefrontal regions, frontal base and medial thalamus during the chronic phase (Figure 3) [4]. However, in our study in a group of patients with chronic mental and cognitive symptoms following mild TBI and without visible brain lesions on MRI, glucose metabolism was significantly reduced across the entire bilateral prefrontal area (Figure 4) [5]. These topographical patterns of glucose hypometabolism are obviously different, and the results suggest that another pathological mechanism distinct from DAI may cause mental and cognitive symptoms in the chronic phase in patients with mild TBI and without visible brain lesions on MRI.

Additionally, our study identified a compensatory increase in glucose metabolism in the limbic system and cerebellum in patients with mild TBI (Figure 4) [5]. This pattern of altered metabolism in patients with mild TBI is topographically similar to that seen in patients with major depressive disorder. Based on these findings, the pathology contributing to mental and cognitive symptoms in the chronic phase of mild TBI is suggested to be similar to that seen in neuropsychiatric diseases, rather than primary axonopathic organic brain damage, as seen with DAI.

## Serotonin discharge & acute symptoms following mild TBI

Based on the results of our studies, we hereby propose a hypothesis of altered biphasic serotonin discharge from the DRN as a potential cause of symptoms following mild TBI. The initial pathology is presumed to be the acute damage to the DRN, which results in an abrupt discharge of a large amount of serotonin. This causes transient symptoms, such as a mild type of ‘serotonin syndrome’, including hypertension, tachycardia, tachypnea, mydriasis, agitation and increased neuromuscular tone. The symptoms are often seen in the acute phase of mild TBI, in addition to other symptoms, headache, neck pain, dizziness and nausea, due to the physical damage of the head and upper cervical region.

## Serotonin deficiency & post-concussion syndrome

During the several weeks after injury, symptoms improve in most cases of mild TBI, such that normal serotonin secretion from the DRN is presumed to be restored. However, in a certain number of cases, prolonged dysfunction of the DRN may occur, which results in decreased serotonin levels and may lead to decreased activity in numerous brain areas controlled by serotonin projections from the DRN. Serotonin deficiency may then disturb normal mood stabilization in the brain in the chronic phase of mild TBI.

Serotonin deficiency activates the mesolimbic dopamine system via dopamine D2 receptors and causes excessive pleasure- and reward-seeking behavior, which may result in addiction to food, sex and drugs. Some forms of hallucinations and delusions can also occur. Additionally, serotonin deficiency activates the mesocortical dopamine system via dopamine D1 receptors and suppresses function in the prefrontal area, resulting in impairment of cognition, memory and attention. These direct and indirect serotonin deficiency-related symptoms may be seen in the chronic phase in patients with mild TBI as part of post-concussion syndrome. These mechanisms may be supported by the results of our ^18^F-FDG-PET studies in mild TBI (Figure 4) [5].

Fann *et al.* reported that selective serotonin reuptake inhibitors improved depressive symptoms as well as cognitive function, with regard to psychomotor speed, cognitive efficiency, flexible thinking and short-term memory, in patients with mild TBI [6,7]. These results also suggest a relationship between the symptoms following mild TBI and serotonin deficiency.

## Conclusion

Results from our recent collision injury analysis and ^18^F-FDG-PET studies led to the hypothesis of altered biphasic serotonin discharge as a potential pathological mechanism of mild TBI [3,5]. Such a hypothesis can be kept in mind as a pathological mechanism for the appearance of symptoms in mild TBI. Further scientific evidence provided by studies using advanced neuroimaging such as serotonin-PET and biochemical studies to monitor serotonin metabolites in the blood and cerebrospinal fluid is needed to verify this hypothesis.

## Future perspective

Verification of this hypothesis may lead to establish strategies for protection of brainstem damage in mild TBI including TBI in sports and whiplash-associated injury in traffic accident, and be a clue of medical treatment against serotonin mal-metabolism which can be started in the early phase of mild TBI to prevent deterioration of post-concussion syndrome.
